# In Vitro Electrochemical Corrosion and Cell Viability Studies on Nickel-Free Stainless Steel Orthopedic Implants

**DOI:** 10.1371/journal.pone.0061633

**Published:** 2013-04-22

**Authors:** Erfan Salahinejad, Mohammad Jafar Hadianfard, Digby Donald Macdonald, Samin Sharifi-Asl, Masoud Mozafari, Kenneth J. Walker, Armin Tahmasbi Rad, Sundararajan V. Madihally, Lobat Tayebi

**Affiliations:** 1 Department of Materials Science and Engineering, School of Engineering, Shiraz University, Shiraz, Iran; 2 Center for Electrochemical Science and Technology, Department of Materials Science and Engineering, Pennsylvania State University, University Park, Pennsylvania, United States of America; 3 Helmerich Advanced Technology Research Center, School of Materials Science and Engineering, Oklahoma State University, Tulsa, Oklahoma, United States of America; 4 Center for Research Excellence in Corrosion, King Fahd University of Petroleum and Minerals, Dhahran, Saudi Arabia; 5 School of Chemical Engineering, Oklahoma State University, Stillwater, Oklahoma, United States of America; University of California, Berkeley, United States of America

## Abstract

The corrosion and cell viability behaviors of nanostructured, nickel-free stainless steel implants were studied and compared with AISI 316L. The electrochemical studies were conducted by potentiodynamic polarization and electrochemical impedance spectroscopic measurements in a simulated body fluid. Cytocompatibility was also evaluated by the adhesion behavior of adult human stem cells on the surface of the samples. According to the results, the electrochemical behavior is affected by a compromise among the specimen's structural characteristics, comprising composition, density, and grain size. The cell viability is interpreted by considering the results of the electrochemical impedance spectroscopic experiments.

## Introduction

Austenitic stainless steels are successfully used in a wide range of applications, including for orthopedic implants in the biomedical field. AISI 316L is commonly a candidate material for orthopedic, orthodontic, and cardiovascular implants, due to adequate biocompatibility, advantageous mechanical properties, and good corrosion resistance, as well as cost effectiveness [1]. However, problems have been found with the medical-grade alloys. The most important problem is the harmful effect of nickel ions released from the implants due to corrosion, wear, and fretting corrosion [2]. These issues have provided a high level of motivation for the further development of nickel-free stainless steels. Nitrogen, as an austenite stabilizer and strengthening agent, is a promising substitute for nickel that is expensive and the source of serious allergic reactions in human skin. As well as nickel-free stainless steel metallic implants, bio-composites produced from nickel-free stainless steels and hydroxyapatite are also noticeable [3]–[5]. In ASTM standards, two nickel-free, nitrogen-containing medical grade stainless steels have been specified: ASTM ID: F2229 and ASTM ID: F2581. In the recent years, a number of *in vitro* and *in vivo* studies have been conducted on the latter alloy, typically from the viewpoints of biocompatibility, osteointegration, and corrosion behavior [6]–[11].

On the other hand, nano-materials have been the subject of widespread research over the past few decades. Nanocrystalline materials are structurally characterized by a large volume fraction of grain boundaries, which may significantly alter their physical, mechanical, and chemical properties in comparison with conventional, coarse-grained, polycrystalline materials [12]. It is well-established that mechanical alloying is a viable process for synthesizing a wide variety of equilibrium and non-equilibrium alloys, including nano-structured and amorphous powders. Mechanical alloying is a solid-state powder processing technique, involving repeated welding, fracturing, and re-welding of powder particles in a high-energy ball mill [13]. After powder processing, a densification process (sintering) is needed to produce a bulk body.

It is known that residual pores in powder metallurgy parts are deleterious to mechanical properties and corrosion resistance [14]. In order to improve densification, a number of strategies including warm compaction, increasing sintering temperature and time, and using additives for liquid-phase sintering are employed. For liquid-phase sintering of stainless steels, various additives like Cu, Sn, Ni, Pt, Ag, Si, Au, B, P, their compounds and alloys have been explored [14], [15]. However, the use of a biocompatible additive in the process of sintering of medical-grade stainless steels, while obviously important, has not been extensively explored.

The biocompatibility of a material is affected not only by the amount and toxicity of its constituent elements, but also by its corrosion resistance. Recently, amorphous-nanocrystalline, nickel-free stainless steel powders with the chemical composition of ASTM ID: F2581 were successfully liquid-phase sintered with a Mn–11.5 wt% Si additive [16]–[18]. However, the corrosion behavior and biocompatibility of this novel material have not been examined to date. This work aims at evaluating the *in vitro* electrochemical corrosion and cell viability behavior of this material via corrosion potential, polarization, electrochemical impedance spectroscopic (EIS), and cell viability experiments. In addition, a correlation is established between the results of the corrosion studies and cell viability.

## Experimental

### 1. Sample preparation and characterization

In this work, nickel-free, medical-grade austenitic stainless steel samples were prepared by a powder metallurgy route as detailed in Refs. [16]–[18]. In brief, amorphous/nanocrystalline stainless steel powders with the nominal composition of ASTM ID: F2581 were synthesized by mechanical alloying and then densified at 1050°C for 1 h by liquid-phase sintering with a Mn–11.5 wt% Si eutectic alloy as the sintering aid. In this paper, the powder metallurgy samples are designated as A, B and C, according to their additive concentration which was 0, 3 and 6 wt%, respectively. The chemical compositions of the samples, considering the contributions of the Mn–Si sintering aids, are listed in Table 1. The obtained specimens were characterized by the Archimedes water immersion method to determine the density and by X-ray diffraction (XRD; a Shimadzu Lab X-6000 spectrometer with Cu Kα radiation) to evaluate the developed phase and crystallite size. The XRD data were analyzed by the Materials Analysis Using Diffraction (MAUD, Version 2.26) program employing the Rietveld refinement to estimate the phase contents and the crystallite size via the Double-Voigt approach.

**Table 1:** Chemical composition of the stainless steel samples (wt%).

Element	A	B	C	316L
Cr	17	16.5	16	17
Mn	10	12.3	14.7	10
Ni	-	-	-	10
Mo	3	2.9	2.8	2
Si	0.4	0.7	1	0.4
C	0.5	0.49	0.47	0.03
N	0.2	0.19	0.18	0.03

### 2. Electrochemical experiments

For electrochemical studies, the sample surface was gradually ground and polished to a mirror-like finish using diamond paste of down to a particle size of 1 µm. The electrochemical behavior of the samples was studied and compared with AISI 316L stainless steel. The experiments were performed in the simulated body fluid (SBF) proposed by Kokubo and Takadama [19] at a pH value of 7.4 under the naturally aerated condition, using a Gamry PC3/300 Potentiostat/Galvanostat/ZRA. The most noticeable feature of the SBF composition from the viewpoint of electrochemical corrosion is the chloride concentration (103 mmol/L). A platinum wire and saturated calomel electrode (SCE) were employed as the auxiliary and reference electrodes, respectively. The exposed surface area of the working electrodes was 0.5 cm^2^.

The open circuit potential (*ocp*) of the samples was recorded in SBF for 48 h, in order obtain a steady-state condition. Afterward, the anodic potentiodynamic polarization curves were obtained at a scan rate of 1 mVs^−1^ from −0.1 V vs. *ocp* to the transpassive potential in the same solution. The impedance measurements were also performed over ten frequency decades from 5 kHz to 10 mHz with an excitation potential amplitude of 10 mV at the *ocp*. All of the electrochemical results were analyzed by using the Gamry Echem Analyst (Version 5.50) software.

### 3. Cytocompatibility studies

Adult human mesenchymal stem cells (hMSC, Lonza Walkersville Inc., MD, USA) were cultured in a mesenchymal stem cell basal medium (MSCGM, Lonza) with other supplements, as recommended by the protocol from Lonza Walkersville. The cells were incubated at 37°C in contact with a 5% CO_2_/95% air atmosphere with the medium being exchanged every 3 to 4 days. The cells proliferated normally with respect to size, shape, and confluency. Upon confluency, the cells were removed from the plate with Clonetics Trypsin-EDTA (Lonza Walkersville). The cells then underwent centrifugation at 300 g for five minutes and were suspended in the growth medium. Viable cell counting was done using the Trypan blue dye exclusion assay. Next, the cells were stained using anamine-reactive, colorless, non-fluorescent dye that diffuses into the cytoplasm of the cells, 5-(and-6)-carboxyfluoresceindiacetate, succinimidyl ester (CFDA-SE) -mixed isomers obtained from Invitrogen Corp. Carlsbad, CA, USA.

All of the stainless steel samples were sterilized by autoclaving at 134°C for 20 min and placed in a 24-well plate pre-coated with bovine serum albumin. Quadruplicate samples were used for each condition. Then, ten thousand cells were seeded onto the tissue culture plastic (TCP) surface and each substrate. A similar number of the cells in suspension were also frozen for the analysis of the initial CFDA-SE content. To achieve a uniform distribution of the cells on the samples, a concentrated (500,000 cells/mL) cell suspension was placed at different locations on each sample and allowed to attach for 30 min prior to adding the growth medium. After one day, the cell-containing samples were fixed in 3.7% formaldehyde for 30 min at room temperature. The samples were dried using ethanol followed by a brief vacuum drying and sputter coated with gold at 40 mA prior to observation under a scanning electron microscope (SEM, Hitachi S-4800).

Cell viability after one day was assessed by two approaches: first, 100 µL of spent media collected on that day were used to analyze cell viability indirectly. Secondly, the specimens containing the cells were washed in MSCGM and cytoplasmic CFDA-SE stained was extracted from the live cells by three cycles of repeated freezing and thawing. The CFDA-SE content in the spent medium and the cytoplasm was assessed by fluorescence intensity in a Gemini XS spectrofluorometer (MDS technologies, Santa Clara, CA) at the excitation and emission wavelengths of 485 nm and 525 nm, respectively. All fluorescence values for the samples were normalized to the TCP for comparison.

## Results and Discussion

### 1. Sample characterization

**Figure 1:**
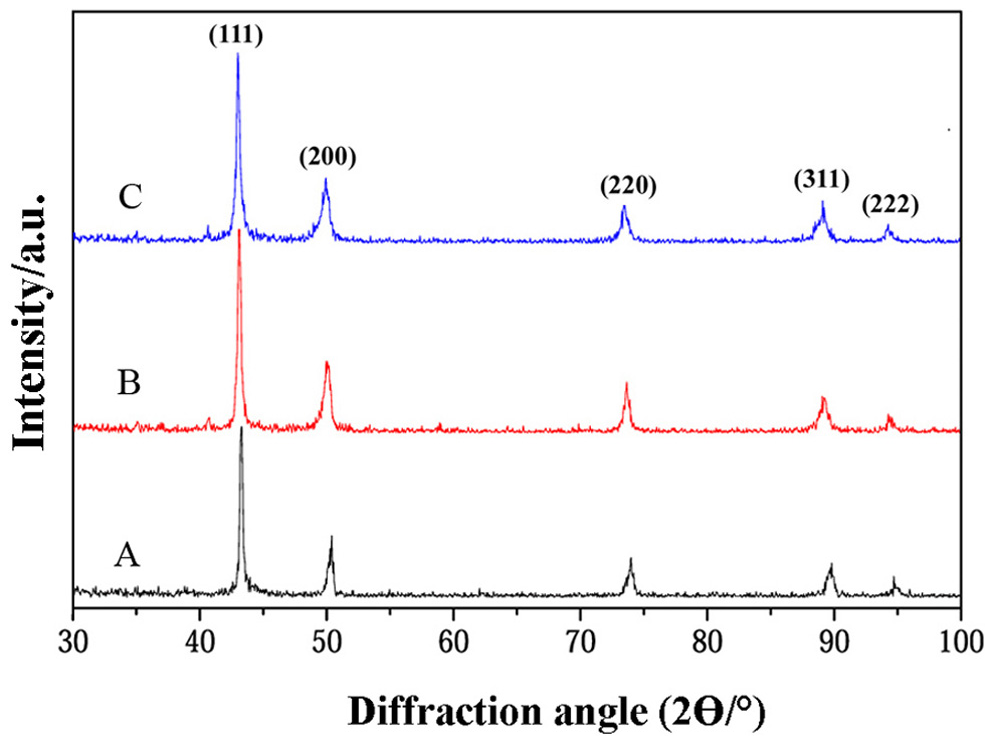
XRD pattern of the nickel-free powder metallurgy stainless steel samples.

Figure 1 shows the XRD pattern of the nickel-free stainless steel specimens sintered with the various contents of the Mn–Si additive. According to the Rietveld analysis conducted by the MAUD program, fully austenitic structures without any second phase were recognized in all the samples. The austenite crystallite sizes determined by the Double-Voigt approach are listed in Table 2, demonstrating the preparation of nanostructured, austenitic stainless steels, as confirmed by transmission electron microscopy [17], [18]. Table 2 also tabulates the mean pore size and relative density of the samples with respect to the theoretical value, measured by the Archimedes water immersion method, indicating an increase in density with increasing the additive content. Indeed, the melted Mn–Si eutectic additive wets the main powder particles, penetrates into particle contacts and pore zones via capillary forces, and provides a path of high diffusivity. This is responsible for a decrease in the pore level and size and thereby an increase in density. It is worth mentioning that, based on X-ray mapping, a uniform distribution of the elements in the structure was realized at a scale comparable with the powder particle size [17], [18].

**Table 2:** Some properties of the stainless steel samples.

Sample	Grain size/nm	Relative density percent	Pore size/µm	PRE
A	20.4±1.1	91.3±1.2	15.0±3.0	34.9
B	22.2±1.4	95.5±2.0	7.0±3.5	33.91
C	25.8±0.8	97.4±1.4	3.5±1.5	32.76
316L	10000	100	-	24.24

### 2. Electrochemical studies

It is well established that, by decreasing the porosity content, the mechanical and corrosion behaviors of powder metallurgy parts are improved [14]. However, in the current work, as well as the positive contribution of the additive to enhancing the sample density, the chemical composition varies and could affect the corrosion behavior. On the other hand, the grain structure, a factor influencing the electrochemical behavior, of the nickel-free samples and 316L is different (Table 2). Although the individual effect of residual porosity, chemical composition, and nanocrystallinity on the electrochemical behavior of materials is known, their simultaneous variation and the detection of the predominant factor are worth studying. To do so, the *in vitro* electrochemical corrosion behavior of the medical-grade stainless steels is focused upon by open circuit potential, anodic polarization, and EIS measurements in SBF.

#### 2.1. Open circuit potential

**Figure 2:**
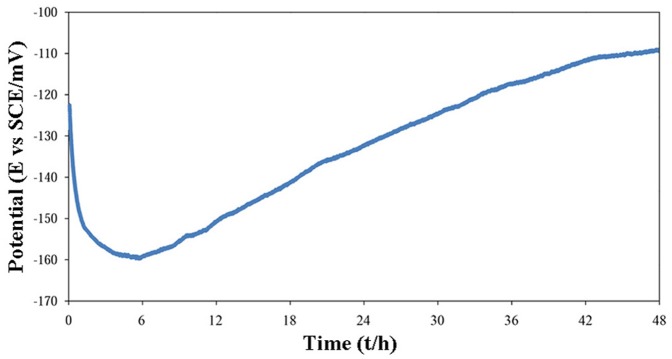
Open circuit potential (*ocp*) vs. immersion time for the sample B.

Typically, the open circuit potential (*ocp*) vs. time behavior for the sample B is shown in Figure 2. The other specimens also showed similar curves in shape, but the potential values were different, as noted below. As can be seen, firstly, the potential decreased and approached a minimum value. Subsequently, it gradually increased and tended towards a steady state value with exposure time up to 48 h. Note that exposure durations beyond 48 h did not change *ocp*. The first decrease in the *ocp* is attributed to removal of the air-formed oxide film on the polished surface. It has been reported that this type of air-formed films is iron oxide-rich [20] and might be degraded in corrosive electrolytes, even in the open circuit condition, either by direct dissolution or via undermining by metal dissolution if the film is not continuous [21]. However, provided that immersion continues for long enough (e.g. 48 h as used in this work), a new film starts to form and varies in thickness, oxidation state, and chemical composition with time. This simple postulate can explain the increase in the *ocp* with time after passing through the minima, where a new surface film is being developed. The same slow increase in the *ocp* with time has been reported for stainless steels previously [20], [22]. The theory of the ennoblement of passive metals upon prolonged exposure under open circuit conditions is now well-developed [23] and it shows that the positive drift in the *ocp* occurs, because of a progressive thickening of the barrier oxide layer. Simultaneously, the corrosion rate is predicted to decrease, as has been observed, at least in the case of Alloy 22 [24]. Comparing the potential at *t* = 0 and at the steady-state potential, it can be concluded that the surface film formed in the solution is more stable and protective than the air-formed film.

After 48 h of immersion, the *ocp* was measured to be -121, -110, -100, and -109 mV (E vs. SCE) for Samples A, B, C, and 316L, respectively. That is, for the nickel-free samples, upon increasing the Mn–Si sintering aid content, more noble corrosion potential values are measured, although based on their chemical composition (Table 1) it might be expected that the surface layer of the alloy with a lower content of the additive is more protective and provides a more noble potential. Under these circumstances, it seems that the residual porosities in the specimens, rather than the chemical composition, play the crucial role in determining the corrosion behavior. The electrolyte in the pores is trapped and stagnates; thus, the pores are converted into preferred sites for localized corrosion attack, especially in chloride-containing solutions, due to a local increase in acidity. This phenomenon could retard the formation and hence degrade the protective characteristic of the surface film. Thus, the pores essentially shift the *ocp* towards more active potentials as they increase the tendency to corrosion attack. Indeed, a compromise between the chemical composition and porosity appears to determine the *ocp*, as noted previously. Potentially, the chemical composition suggests a decrease in the potential by increasing the sintering aid, but the pore suggests an increase in the potential. As the experimental results showed an increase in the *ocp* with the additive content, it is clearly concluded that the contribution of the pores prevails over that of the composition. However, although 316L is more dense than Specimen C, the more active potential of 316L shows the domination of chemical composition in this comparison. On the other hand, the nano-scale structure of Sample C compared with the coarse-grained structure of 316L could improve the protective properties of the surface film and may increase the *ocp*. The effect of the constituent alloying elements and nano-sized structure on the corrosion behavior is discussed later.

On the whole, although the *ocp* studies present an initial and qualitative classification of the corrosion attack, specific to the experimental conditions of the test, the polarization and impedance approaches allow more accurate evaluations of localized and general corrosion, as focused on below.

#### 2.2. Anodic potentiodynamic polarization

**Figure 3:**
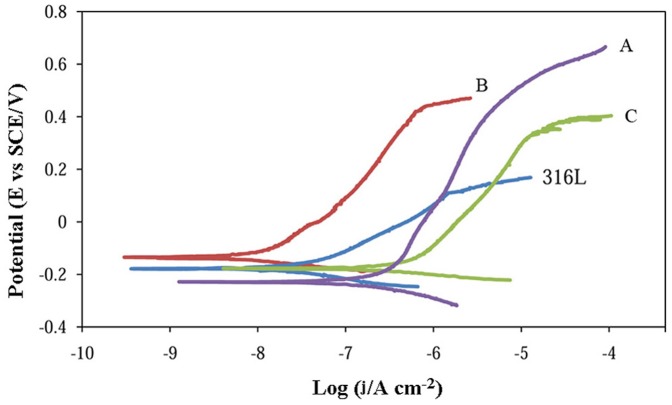
Potentiodynamic polarization curves for the stainless steel samples.

After immersion of the specimens in SBF for 48 h and the attainment of steady-state conditions, anodic potentiodynamic polarization scans were carried out (Figure 3), in order to assess the degree of passivation and to characterize the localized corrosion behavior. The ease of formation and the stability of the protective passive film can be realized from the polarization results (passive current density and passive range).

The passive current density, which is an inverse indicator of passivity, increases in this order: B<316L<A<C. In the electrochemical behavior, three properties of the steels are of relevance: chemical composition, density, and grain size. The lower current density of B than that of 316L shows the domination of the chemical composition (especially N and Mo) and grain structure, not porosity level. The better behavior of 316L, compared with A and C, is indicative of the contribution of the porosity level, which results in a higher passive current density in the case of the latter. As noted above, the presence of pores in the structure could affect the formation of a protective passive surface film in the course of polarizing and lead to localized corrosion processes that would contribute to the anodic current via oxidation of alloy within the pores. As well as these effects, a few studies have noted that the critical current density for passivation increases with increasing nitrogen content of the stainless steels [25], [26], which may make a contribution to the lower passive current density of 316L compared with Alloys A and C. Finally, the higher current density of Alloy C compared with Alloy A, is due to the effect of the chemical composition, typically the deleterious role of the Mn content [27], [28].

The passive film stability, estimated from the breakdown potential and the width of the passive range, increases in this order: 316L<C<B<A. This trend clearly reflects the dependency of the breakdown potential and passive range on the composition and grain size, not density. The latter behavior is in good agreement with pitting resistance equivalent numbers (PRE) listed in Table 2, calculated by the following equation [29]:
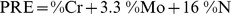
(1)where %Cr, %Mo, and %N are the weight percentage of Cr, Mo, and N in the alloys respectively. The better pitting resistance of the nickel-free alloys than 316L is mainly due to the contribution of N resulting in this considerable difference in the PRE and also in their grain size. The positive role of Mo in the pitting resistance is also noteworthy. For the powder metallurgy samples, the degradation of the pitting resistance with increasing Mn–Si sintering aid content is due to the decrease of the PRE (i.e. the roles of Cr, Mo, and N) and to the increase in the Mn content (see Table 2 and Eq. 1). Mn affects disadvantageously the chemical composition and characteristics of the passive layer and has a deleterious influence on the corrosion resistance [27], [28].

There have been many mechanisms proposed to explain the beneficial effects of N alloying on the corrosion behavior of stainless steels, including ammonia production, surface enrichment, anodic segregation, salt film formation, and synergistic effects involving Mo, N, and Cr [30]. Typically, N improves the pitting resistance in aqueous chloride solutions, since it is concentrated at the surface and is believed to produce ammonia, which neutralizes hydrogen ions that are produced in incipient crevices via differential aeration. Thus, even in neutral and alkaline solutions, a locally acidic environment is created inside pits and/or pores, due to the hydrolysis of dissolved metals. In addition, N, in combination with Mo, produces a more protective passive film, since both are thermodynamically more noble than Fe and their dissolution reaction is a slow and multi-electron process [31].

On the other hand, nanocrystallization has a beneficial role in the electrochemical corrosion behavior, when the corrosion products are insoluble; for example, for stainless steels. It has been reported that nanocrystallization alters the composition, morphology, and growth process of the passive film, which could improve its compactness and protective properties [21], [32].

#### 2.3. Electrochemical impedance spectroscopy

**Figure 4:**
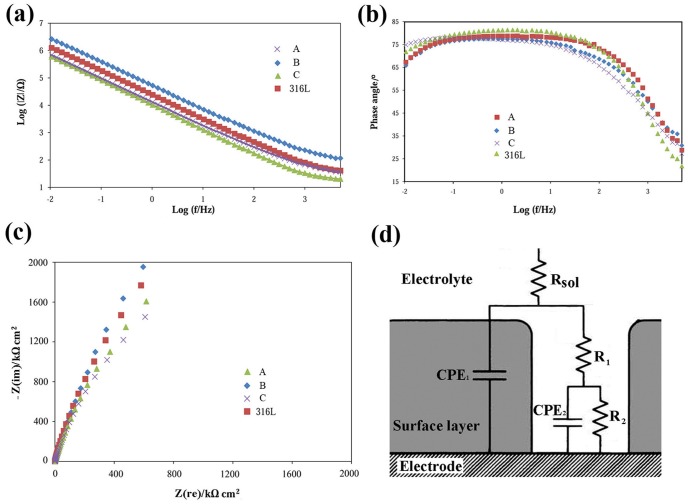
Bode impedance (a), Bode phase angle (b) and Nyquist (c) plots; and equivalent electrical circuit used for the quantitative analyses of the impedance spectra (d).

Nyquist and Bode impedance plots for the samples at the *ocp* after immersion in SBF for 48 h is indicated in Figure 4. It can be seen that all of the samples display a similar impedance behavior under the chosen test conditions. The Nyquist diagrams are typical of passive systems, with large values for the real and imaginary components of the impedance at low frequencies. A broad plateau can be seen in the Bode phase angle plots. According to these curves, the impedance values infer that the corrosion resistance increases in this order: C<A<316L<B. This is consistent with the order observed for the passive current density (Figure 3). Considering the impedance spectral features and the nature of the surface oxide film formed due to immersion in SBF for 48 h, an equivalent electrical circuit (Figure 4d) was used to quantitatively analyze the impedance spectra. Fitting of the model to the data was accomplished by using a nonlinear least-squares method, resulting in the low values of ‘goodness of fit (residual error)’ (Table 3). Note that fitting was not successful when the passive layer was ignored in the modeling; that is, when only a parallel combination of double layer capacitor/space charge capacitance and charge transfer resistance was considered. This effectively verifies the presence of a surface passive film affecting the corrosion behavior. Due to the distribution of relaxation times resulting from inhomogeneities at the electrode surface at nano/micro scale (like roughness, porosity, adsorption, and/or diffusion) [33], [34], the use of constant phase elements (CPEs), rather than pure capacitances, improved the fitting. The impedance (*Z_CPE_*) of a CPE is expressed as:
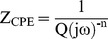
(2)where *Q* is the capacitance in F, *ω* is the angular frequency (*ω* = 2π*f*; *f* is the frequency in Hz), *j* is (−1)^0.5^, and *n* is an empirical exponent that is less than one and describes the deviation from an ideal capacitor (*n* = 1, 0, −1, and 0.25–0.5 corresponding to a pure capacitor, resistor, inductor, and Warburg impedance, respectively). Therefore, the total impedance of the proposed circuit is:
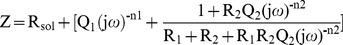
(3)where *R_sol_* is the electrolyte resistance, *R_1_* is the pore resistance or the resistance of ion conducting paths in the film, and *R_2_* is the charge transfer resistance. CPE_1_ (*Q_1_*, *n_1_*) and CPE_2_ (*Q_2_*, *n_2_*) correspond to the distributed capacitors of the film and double layer, respectively.

**Table 3:** Equivalent electrical circuit parameters obtained by the EIS studies.

Sample	*R_1_*/Ω cm^2^	*Q_1_*/µΩ^−1^ s^n1^ cm^−2^	*n_1_*	*R_2_*/MΩ cm^2^	*Q_2_*/µΩ^−1^ s^n1^ cm^−2^	*n_2_*	Residual error
A	196.5±5.1	6.3±0.2	0.91±0.02	3.3±0.1	6.7±0.3	0.89±0.04	6.2 * 10^−5^
B	302.0±7.2	5.0±0.8	0.86±0.04	8.1±0.3	5.4±0.6	0.90±0.04	9.1 * 10^−5^
C	124.7±4.7	6.6±0.4	0.92±0.02	2.8±0.1	7.8±0.4	0.89±0.05	5.8 * 10^−5^
316L	290.5±7.1	5.9±0.7	0.91±0.04	5.5±0.2	5.8±0.5	0.92±0.06	8.0 * 10^−5^

The equivalent circuit parameters are listed in Table 3. According to Table 3, since *R_1_* is significantly lower than *R_2_*, polarization resistance (*R_p_*), which is the sum of all the ohmic resistances, can be considered to correspond to *R_2_*. *R_p_* is the surface's total resistance to general corrosion and can be regarded as a criterion for judging the corrosion rate and ion release rate: the higher *R_p_*, the lower corrosion rate under the test conditions. Thus, the corrosion rates and ion release rates of the samples show an increasing trend in this order: B<316L<A<C, which can be justified by the previous discussions presented above for the polarization behavior. It is worth mentioning that a higher pore content provides a larger exposed real. Since the film resistance is calculated with respect to the apparent surface area, this point may challenge the comparisons and thereby the discussions previously presented. However, as realized below in the cell viability, which merely depends on the ion release rate, this effect does not discredit the interpretation of the data and the conclusions remain valid.

### 3. Cell viability

**Figure 5:**
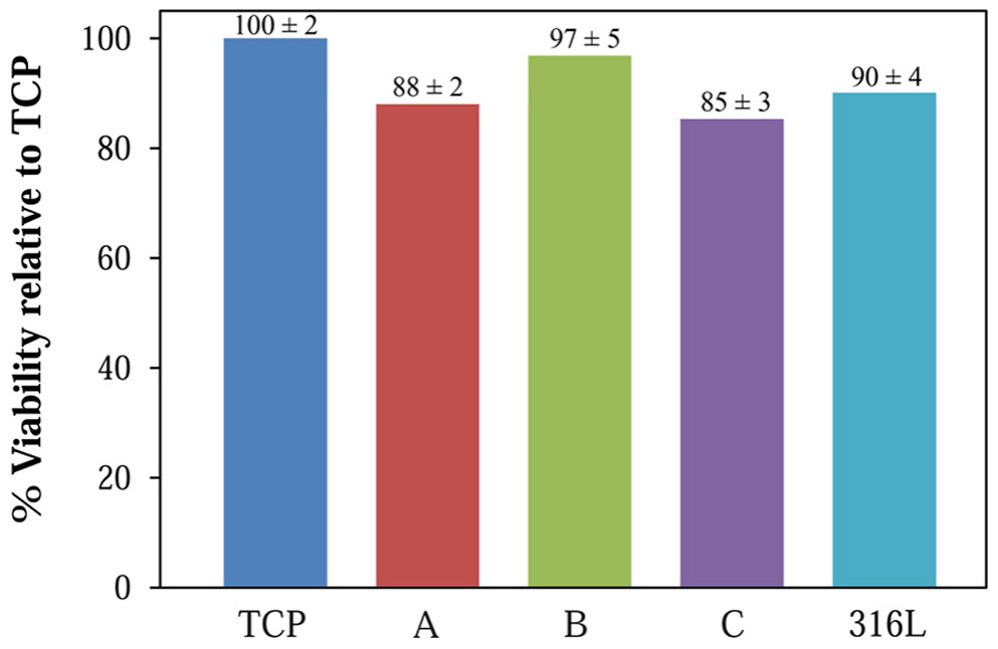
Cell viability on the TCP and stainless steels after one day, determined by fluorescence in the cytoplasmic extract.

It has been previously pointed out that cell viability can be accurately assessed by pre-staining the cells with CFDA-SE [35]. Using this approach, the viability of hMSCs on the stainless steel specimens was evaluated after one day of culture. According to Figure 5, the cytoplasmic contents obtained from the freeze/thaw cycle indicate that the cell viability for the stainless steel samples is considerable and is comparable to TCP, suggesting that they are not toxic to the cells. The supernatants collected after one day also demonstrated similar trends. It can be seen that the viability displays a slight decline in this order: B>316L>A>C.

**Figure 6:**
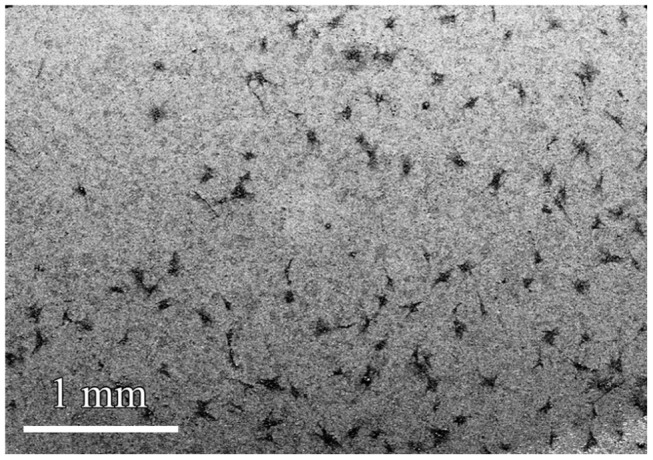
Low-magnification SEM micrograph of cells fixed on the sample B.

**Figure 7:**
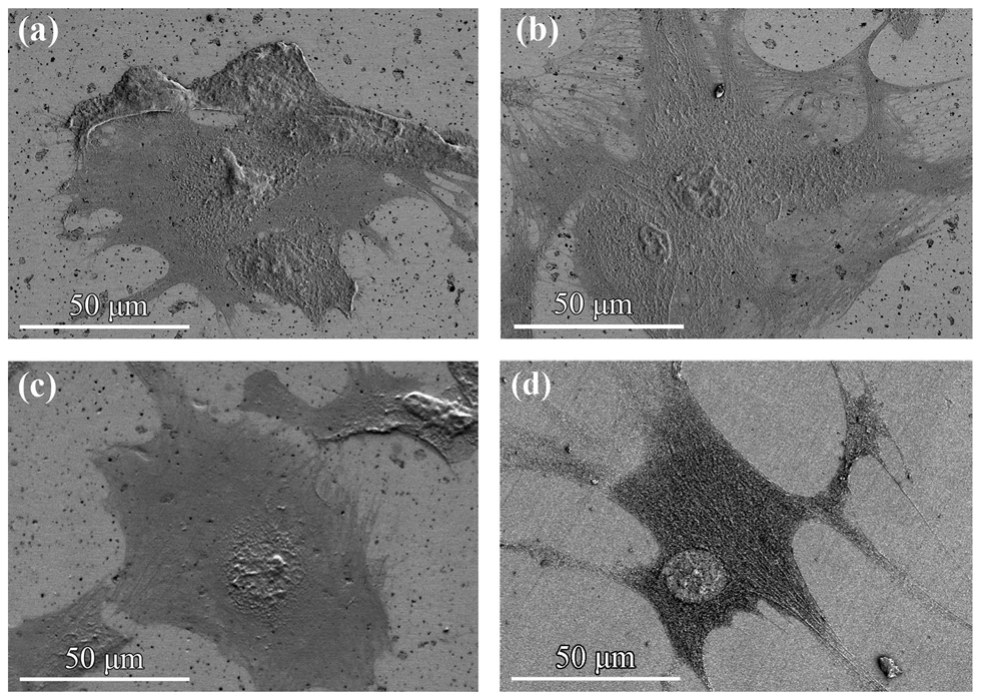
SEM micrograph of cells fixed on A (a), B (b), C (c), and 316L (d).

Figure 6 shows a low-magnification SEM micrograph of cells fixed on the stainless steel sample B, presenting a desirable distribution and advantageous features of the cells on the surface. The SEM micrograph of the other samples depicted similar features. The cell's morphology and spreading are more obvious in the high-magnification SEM micrograph of the cell cultured surfaces (Figure 7). According to the SEM micrographs, the cells are well-spread on all of the surfaces with numerous lamellipodia and filopodia. The body of the cells has a mean size of 20 µm and their majority developed arm-like cytoplasmatic extensions up to 100 µm in length. This desirable feature is indicative of good cellular migration and attachment, implying the biocompatibility of the implants, as realized by the above, quantitative analysis.

The minor difference in the cell adhesion behavior can be explained by the amount of released metal ions. A consideration of the results of the electrochemical experiments implies that the polarization resistance estimated by impedance spectroscopy presents a ranking that is identical to the quantitative cell adhesion assessment. It is reasonable to correlate the results of these tests, because the impedance measurements (especially in terms of the polarization resistance) report a tendency toward general corrosion and hence toward an increasing amount of released metal ions, which are the essential factors affecting the cell viability. Thus, the similarity in the obtained ranking infers that the impedance evaluation is a suitable approach for analyzing the cell viability, at least qualitatively. Note that the cell adhesion directly deals with the ion release rate, not the real surface area, as noted in the impedance part of this paper. Hence, the connection of the sample ranking, based on the impedance and cell viability studies, reflects the fact that in this study the real surface area, including that of the surface pores, does not govern in determining the ranking via the impedance analyses, as conducted in this study.

## Conclusions

The *in vitro* electrochemical corrosion and cell viability behaviors of nanostructured nickel-free stainless steels prepared by the powder metallurgy process were compared with AISI 316L. For briefness, the powder metallurgy alloys were designated as A, B, and C which had 0, 3, 6 wt% of the Mn-Si additive used for liquid phase sintering. The following conclusions can be drawn from this work:

1. By increasing the sintering aid content and thereby lowering the porosity level, the corrosion potential increased. The corrosion potential of 316L was close to that of Sample B.
2. The passive current density showed a dependency on the composition, density, and grain structure. The passive current density ranked in this order: B<316L<A<C.
3. The pitting resistance of the samples followed the trend predicted by the PRE numbers: with the highest resistance for the sample sintered without the additive and the lowest for 316L.
4. The polarization resistance, estimated from the impedance measurements (film resistance), decreased in this order: B>316L>A>C.
5. The study of cell culturing for 1 day on the samples showed their biocompatibility and obeyed the rank obtained from the impedance evaluations.
